# Antioxidant Activity and Toxicity of Fullerenols via Bioluminescence Signaling: Role of Oxygen Substituents

**DOI:** 10.3390/ijms20092324

**Published:** 2019-05-10

**Authors:** Ekaterina S. Kovel, Anna S. Sachkova, Natalia G. Vnukova, Grigoriy N. Churilov, Elena M. Knyazeva, Nadezhda S. Kudryasheva

**Affiliations:** 1 Institute of Biophysics SB RAS, FRC KSC SB RAS, 660036 Krasnoyarsk, Russia; n-qdr@yandex.ru; 2 Institute of Physics SB RAS, FRC KSC SB RAS, 660036 Krasnoyarsk, Russia; Nata_hd@rambler.ru (N.G.V.); churilov@iph.krasn.ru (G.N.C.); 3 National Research Tomsk Polytechnic University, 634050 Tomsk, Russia; asachkova@tpu.ru (A.S.S.); knyazeva@tpu.ru (E.M.K.); 4 Siberian Federal University, 660041 Krasnoyarsk, Russia

**Keywords:** bioactive compound, fullerenol, antioxidant activity, toxicity, reactive oxygen species, bioluminescence bioassay

## Abstract

Fullerenols are nanosized water-soluble polyhydroxylated derivatives of fullerenes, a specific allotropic form of carbon, bioactive compounds, and perspective basis for drug development. Our paper analyzes the antioxidant activity and toxicity of a series of fullerenols with different number of oxygen substituents. Two groups of fullerenols were under investigation: (1) C_60_O_y_(OH)_x_, C_60,70_O_y_(OH)_x_, where x+y = 24–28 and (2) C_60,70_O_y_(OH)_x_, Fe_0,5_C_60_O_y_(OH)_x_, Gd@C_82_O_y_(OH)_x_, where x+y = 40–42. Bioluminescent cellular and enzymatic assays (luminous marine bacteria and their enzymatic reactions, respectively) were applied to monitor toxicity in the model fullerenol solutions and bioluminescence was applied as a signaling physiological parameter. The inhibiting concentrations of the fullerenols were determined, revealing the fullerenols’ toxic effects. Antioxidant fullerenol’ ability was studied in solutions of model oxidizer, 1,4-benzoquinone, and detoxification coefficients of general and oxidative types (*D_G_*_T_ and *D_OxT_*) were calculated. All fullerenols produced toxic effect at high concentrations (>0.01 g L^−^^1^), while their antioxidant activity was demonstrated at low and ultralow concentrations (<0.001 g L^−^^1^). Quantitative toxic and antioxidant characteristics of the fullerenols (effective concentrations, concentration ranges, *D_G_*_T_, and *D_OxT_*) were found to depend on the number of oxygen substituents. Lower toxicity and higher antioxidant activity were determined in solutions of fullerenols with fewer oxygen substituents (x+y = 24–28). The differences in fullerenol properties were attributed to their catalytic activity due to reversible electron acceptance, radical trapping, and balance of reactive oxygen species in aqueous solutions. The results provide pharmaceutical sciences with a basis for selection of carbon nanoparticles with appropriate toxic and antioxidant characteristics. Based on the results, we recommend, to reduce the toxicity of prospective endohedral gadolinium-fullerenol preparations Gd@C_82_O_y_(OH)_x_, decreasing the number of oxygen groups to x+y = 24–28. The potential of bioluminescence methods to compare toxic and antioxidant characteristics of carbon nanostructures were demonstrated.

## 1. Introduction

Carbon nano-objects are of great interest for different fields of medicine, pharmacology, and biotechnology due to their specific biological activity [1,2,3]. It is known that high-dose exposures to bioactive compounds can inhibit physiological functions of multiple organisms and, hence, produce toxic effects, while low-dose exposures can activate the physiological functions due to optimization of complex metabolic processes [4].

**Scheme 1:**
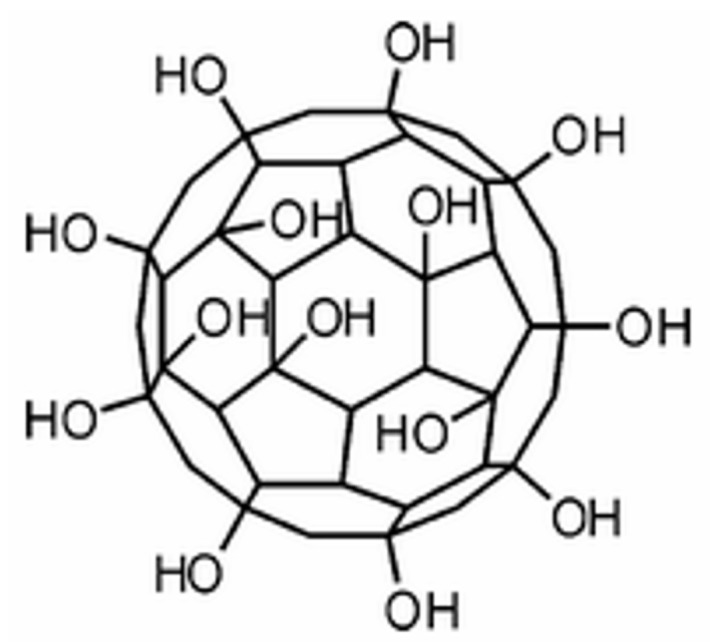
Hypothetical structure of fullerenol C_60_.

Fullerenols (F) are known to be rigid nanosized carbon particles, and are water-soluble polyhydroxylated derivatives of fullerenes. Scheme 1 presents the hypothetical structure of F with 60 carbon atoms as an example.

Similar to fullerenes, fullerenols are electron-deficient structures and this property makes them efficient catalyzers in biochemical reactions, as well as in prospective medical drugs. Fullerenols are amphiphilic structures: “hydroxyl groups provide them with aqueous solubility, while the fragments of fullerene skeleton—with affinity to hydrophobic enzymatic fragments and lipid structures of cellular membranes” [1,2]. Amphiphilic properties and antiradical activity provide a wide range of fullerenols’ biological effects: from neutralization of free radicals [5] to cell protection and drug transportation [1,5,6,7]. Fullerenols can be used in radiobiology, chemotherapy, and neurology [5,6], ensuring an important alternative to conventional pharmaceuticals. The antioxidant properties endow fullerenols with the ability to neutralize reactive oxygen and nitrogen species [8,9,10,11,12], and to function as radioprotectors [10], antitumor [13], or neurological [5,10,11,12] drugs.

Structural properties influence the biological activity of fullerenols. Hydroxyl substituents distort the π-electron system conjugation of the fullerene skeleton, change the electron-acceptor ability of the nanoparticles, and, hence, can affect their catalytic activity. This can result in different toxicity and antioxidant activity of fullerenols with different number of hydroxyl substituents.

Biological activity of C_60_-fullerenols with different number of hydroxyl groups have been studied over the last decades [5,6,7,8,9]. Toxic and antioxidant effects of fullerenols were revealed. However, different biological test objects did not provide a comparability of the fullerenol biological activity. Comparable conditions were provided by Eropkin and co-authors [14]. They studied the biological activity of a series of fullerenols, C_60_(OH)_12–14_, C_60_(OH)_18–24_, and C_60_(OH)_30–38_. It was found that C_60_(OH)_12–14_ was insoluble in water and did not show biological activity, while C_60_(OH)_18–24_ was soluble and showed maximum antiviral and protective properties. The nitric oxide-scavenging and protective activity of C_60_(OH)_18–24_ was demonstrated in [5,8]. However, in spite of extensive studies of fullerenol biological activity, dependencies of their toxic and antioxidant characteristics on concentrations were not revealed and compared. 

Hydration of C_60_-fullerenol with different number of hydroxyl groups (from 8 to 44) was theoretically studied in [15]. It was proved that hydration of C_60_(OH)_44_ was less effective despite the large number of hydroxyl groups. The authors concluded that the involvement of >36 hydroxyl groups to the fullerenol structure resulted in effective intramolecular interactions of OH-groups, conflicting with the hydrogen bonds with the solvent.

Since an increase of a number of hydroxyl substituents reduces the available π-electron system conjugation, it can reduce fullerenols’ ability of reversible radical trapping [14]. This might be a reason for the variation in content of reactive oxygen species (ROS) in aerated aqueous solutions, with effects on the following biological structures: cells, enzymes, low-molecular components, etc. 

It is known that ROS groups include a number of free oxygen radicals or radical precursors, such as semiquinones, superoxide anion-radical (•O_2_^−^), hydroxyl (•OH) and peroxide (HOO•) radicals, hydrogen peroxide (H_2_O_2_), peroxide anion (HOO^−^), singlet oxygen (^1^O_2_) [16], hypochloric acid (HOCl), peroxynitrite radical (ONOO-), and others. They are formed in cells as natural products of oxygen metabolism, their content is labile, and they can initiate formation of additional radicals. ROS play a role as mediators of important intracellular signaling pathways [17], thereby regulating cellular processes (respiration, division, etc.), inducing the immune system, mobilizing ion transport systems, and triggering programmed cell death (apoptosis) [18]. 

Fullerenols’ catalytic activity is due to electron donation/acceptance and oxygen radical scavenging. For example, the process of O_2_^•−^- neutralization can be presented as follows:

$$2O_{2}^{•-}+2H_{2}O\overset{\;F\;}{\to }H_{2}O_{2}+2OH^{-}+O_{2}^{}$$

where F plays the role of a catalyzer. A detailed description of possible mechanisms of oxygen radicals’ neutralization is presented in [11]. 

As the ROS impact on living organisms is evident [19,20,21,22,23,24,25,26,27], a study of ROS content in fullerenol’ solutions can elucidate the mechanism of fullerenol’ biological effects. 

Our paper used the physico-chemical approach to study the properties of bioactive compounds. This approach is based on the “structure-function” relations and allows for predicting the toxic and antioxidant properties of fullerenols. The relations can help in further applied studies, from synthesis to medical adaptation. Additionally, the relations can help to minimize further routine experiments with organs and whole organisms, which are usually time-consuming, expensive, and less reproducible.

The physico-chemical approach assumes an application of simple biological assays as models. We applied two types of bioassays of different levels of organization—cellular and enzymatic. Both bioassays used luminescence intensity as a test parameter. Luminescent registration provides high rates, low costs, and convenience of the bioassay procedures; it ensures simultaneous multiple analyses and, hence, proper statistical processing and reliability for the results of biomedical investigations. 

Cellular bioassay is based on luminous marine bacterium. This bioassay is classic and has been widely used for more than five decades [28,29,30,31,32,33]. Enzymatic bioluminescence assay progressed from early 90s [29,34,35,36]. Solid immobilized bacterial and enzymatic preparations are developed now as a basis for bioluminescent biosensors [34,37,38,39,40]. The bacterial and enzymatic bioassays are tools for investigation of the toxic mechanisms at the cellular and molecular levels, respectively. Classification of the toxic effects was suggested first in [41] and developed later in [42,43,44,45]. It describes the (1) physicochemical, (2) chemical, and (3) biochemical basis for the toxic effects in the bioluminescence assay systems. 

Antioxidant properties of bioactive compounds provided a new perspective for biosensor applications [46,47]. The bioassay systems, based on the luminous bacterium or its enzymatic reactions, are proper candidates for this application. Both assays can evaluate a general toxicity in the test samples under conditions of the oxidative stress. Additionally, the enzymatic bioassay is specific to oxidizers [45] and can be applied to monitor the oxidative toxicity in the solutions. This type of toxicity is attributed to the redox properties of the toxic compounds only, while the other toxicity type, general toxicity, integrates all interactions of toxic compounds with the bioluminescent assay system: redox reactions, polar and non-polar interactions, etc. Previously [48,49], we evaluated the general and oxidative toxicities in solutions of inorganic and organic oxidizers (polyvalent metals and quinones) using the bioluminescent enzymatic assay system. Changes in general toxicity and oxidative toxicity under exposure to humic substances (bioactive compounds of natural origination, products of organics decomposition in soils) were studied in [50,51,52]. The bioluminescence technique for evaluating the antioxidant activity of bioactive compounds was described in [53,54,55]. Antioxidant properties of two fullerenols were studied for the first time and compared using bioluminescence enzymatic technique in [53].

The current study develops a fundamental basis for fullerenol medical application. We review, analyze, and compare our new and previous experimental results, obtained under comparable conditions. Our paper analyzes antioxidant activity and toxicity of a series of fullerenols with different numbers of oxygen substituents. Chemical formulas of the fullerenols and their short abbreviations are presented in Table 1. The fullerenols were attributed to two clusters involving either 24–28 or 40–42 oxygen substituents (F1, F2 or F3, F4, F5, respectively, Table 1). Enzyme-based and cellular-based luminescent bioassays were applied to evaluate toxicity and antioxidant properties of the fullerenols. Ranges of toxic and antioxidant concentrations of fullerenols were of particular attention. Quantitative antioxidant characteristics (detoxification coefficients) are presented in a wide range of fullerenol concentrations. Possible antioxidant mechanisms and the role of ROS in the effects of the fullerenols are discussed. Toxic effect of a perspective endohedral metal-fullerenol with gadolinium atom involved (F5, Table 1) is evaluated. A recommendation is provided for the synthesis of new low-toxic endohedral gadolinium-fullerenol preparation.

## 2. Results and Discussion

### 2.1. Toxicity and Antioxidant Activity of Fullerenols via Bioluminescent Assays

#### 2.1.1. Fullerenol Toxicity

We examined the toxicity factor of fullerenols with different number of oxygen groups using cellular and enzymatic bioluminescence assays. Suppression of bioluminescence intensity was considered to be evidence of fullerenol toxic effect. This suppression is concerned with the inhibition of membrane and intracellular processes (for bacterial cells), or chemical and biochemical reactions (for enzyme system).

**Figure 1:**
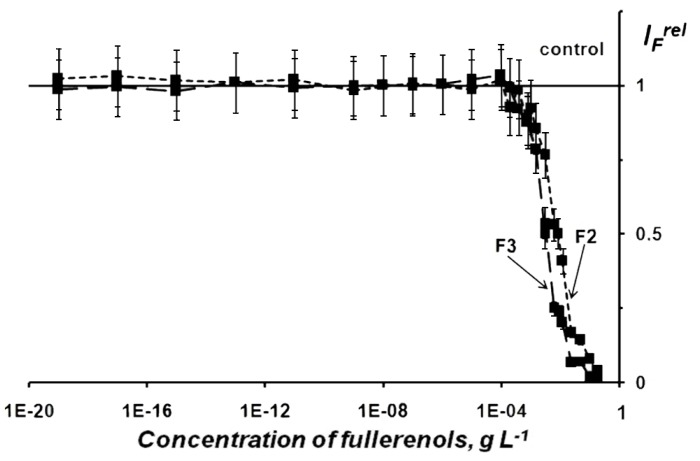
Bioluminescence intensity, *I^rel^*, at different concentrations of fullerenols F2 and F3 for the cellular assay.

**Figure 2:**
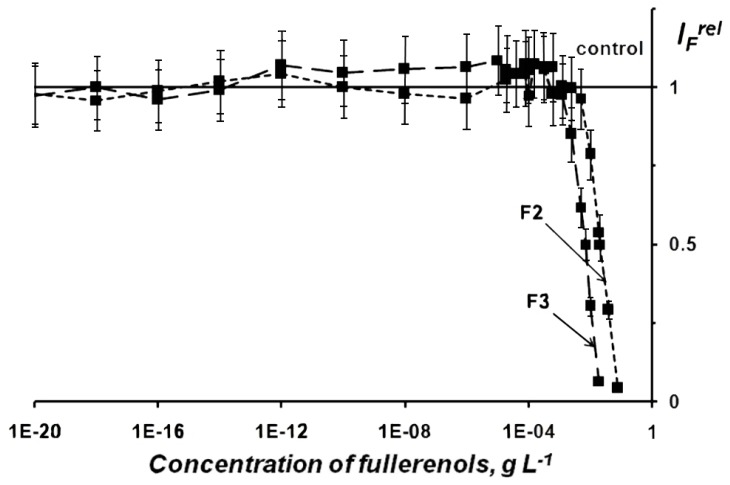
Bioluminescence intensity, *I^rel^*, at different concentrations of fullerenols F2 and F3 for the enzymatic assay.

Dependencies of relative bioluminescence intensities $I_{F}^{rel}$ (Equation (1)) on the concentration of the fullerenols were obtained. Examples of these dependencies are presented in Figure 1 and Figure 2. Fullerenols F2 and F3 were chosen as nanostructures with similar carbon carcasses, but different number of oxygen substituents. It is seen that F2 and F3 suppressed bioluminescence of the cellular assay at concentrations >0.002 g·L^−1^ and >0.001 g·L^−1^ (Figure 1), and suppressed bioluminescence of the enzymatic assay at concentrations >0.010 g·L^−1^ and >0.003 g·L^−1^ (Figure 2), respectively. The results demonstrate the higher toxicity of F3, i.e., fullerenol with more oxygen substituents.

Fullerenol’ effective concentrations *EC*_50_ were determined and are presented in Table 2. It is evident from this Table that F1 and F2 (i.e., fullerenols with lower oxygen numbers) were characterized by higher *EC*_50_ values and, hence, produced lower toxicity effects, as compared to fullerenols F3, F4, and F5. The *EC*_50_ value of F4 in the bacterial assay (0.021 g L^−1^, Table 2) was an exception. Probably, iron atoms, involved in the fullerenol preparation, specifically affected the metabolism of the bacterial cells, according to their microelement properties.

Toxicity of the new endohedral metal-fullerenol F5 (gadolinium atom inside the fullerenol carbon carcass) was studied using the enzyme-based bioassay, Table 2. 

It is known that gadolinium-based preparations have potential in magnetic resonance imaging and cancer research due to gadolinium’ unique paramagnetic properties [56]. However, the high toxicity of gadolinium-based chemotherapeutic drugs limits their clinical application. They are known to lead to severe skin and systemic diseases, renal dysfunction [57], and intracranial deposition of gadolinium [58]. The problem of toxicity of gadolinium preparations might be solved by involvement of gadolinium into the fullerenol carcass. An endohedral gadolinium-fullerenol preparation Gd@C_82_O_y_(OH)_x_ where x+y = 40–42, was synthesized as described in Section 3 (Materials and Methods). However, our preliminary study demonstrated a moderate toxicity of this compound (F5, EC_50_ = 0.018 g L^−1^, Table 2), and it is desirable to reduce this toxicity. Since the results of the current study predicted lower toxicity of the preparations with fewer oxygen substituents, we can recommend synthesis of endohedral gadolinium-fullerenol preparations with fewer oxygen groups: x+y = 24–28 or lower, as a development of these investigations. Bioluminescence toxicological monitoring can provide a prompt toxicity evaluation of the newly synthesized gadolinium preparations at cellular and biochemical levels (bacteria- and enzyme-based bioassays, respectively).

#### 2.1.2. Fullerenol Antioxidant Activity

To compare the antioxidant activity of fullerenols, we chose a range of fullerenol concentrations providing the absence of the fullerenol inhibiting effect. These concentration ranges (CR) are presented in Table 2 for both cellular and enzymatic assays.

The antioxidant ability of fullerenols was studied using cell-based and enzyme-based assays (luminous marine bacteria and their enzymatic reactions, respectively). The bioassays were applied to monitor toxicity changes in solutions of the model oxidizer 1,4-benzoquinone under additions of the fullerenols and bioluminescence was applied as a signaling physiological parameter. Changes in toxicities of the general or oxidative type (*GT* or *OxT*, respectively) were evaluated, and detoxification coefficients *D_GT_* or *D_OxT_* were calculated. Values of *D_GT_* > 1 and *D_OxT_* > 1 revealed a toxicity decrease under the exposure to fullerenols, i.e., detoxification of solutions of the oxidizer.

##### Change in General Toxicity (GT) of Oxidizer Solutions Under Exposure to Fullerenols

**Figure 3:**
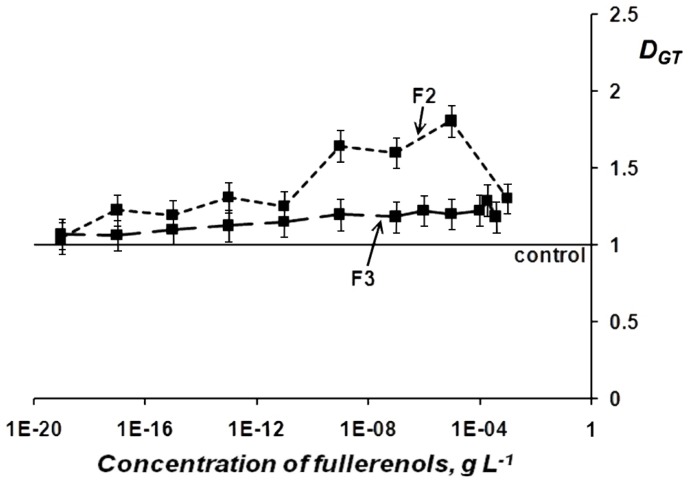
Detoxification coefficients *D_GT_* vs. concentration of fullerenols F2 and F3 in solutions of 1,4-benzoquinone (2.5∙10^−7^ M) for the cellular assay.

**Figure 4:**
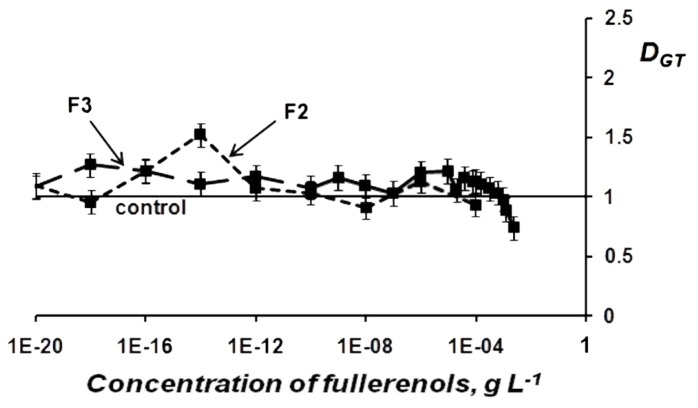
Detoxification coefficients *D_GT_* vs. concentration of fullerenols F2 and F3 in solutions of 1,4-benzoquinone (10^−4^ M) for the enzyme-based assay.

Bioluminescence intensity in the cellular and enzymatic systems was measured in solutions of the model oxidizer, 1,4-benzoquinone (at *EC*_50_), in the absence and presence of fullerenols. Concentrations of the fullerenols varied in a wide range as shown in Figure 3; Figure 4 for bacterial and enzymatic assays, respectively. Detoxification coefficients *D_GT_* were calculated according to Equation (2). The results are presented in Figure 3 and Figure 4, with F2 and F3 taken as examples. As discussed before [50], the difference in the responses of cells and enzyme reactions can be attributed to the active role of the bioassay systems in the detoxification processes. Antioxidant activity of low-concentration fullerenol solutions was discussed previously [54] in terms of the hormesis phenomenon [4]. 

Cellular assay (Figure 3) showed that the oxidizer’ solutions were detoxified (*D_GT_* > 1) in the concentration ranges of 10^−19^–10^−3^ and 10^−15^−4 × 10^−4^ g L^−1^ for F2 and F3, respectively. Maximal values of *D_GT_* were about 1.8 and 1.3, respectively.

Enzyme-based assays showed lower values of *D_GT_*, when compared with the cell-based assay. Figure 4 demonstrates the detoxifying effect of F2 and F3 in 1,4-benzoquinone solutions (*D_GT_* > 1) in the concentration ranges of 10^−19^–10^−3^ and 10^−20^−10^−10^ g L^−1^, respectively. Maximal values of *D_GT_* were about 1.5 and 1.3, respectively.

The maximal values of D_GT_ of fullerenols F1, F2, F3, and F4 obtained with enzyme and bacterial assays are brought together in Table 3. It is evident that higher D_GT_ values were observed for F1 and F2. This result demonstrated that fullerenols with fewer oxygen groups were characterized by higher detoxifying ability.

##### Change in Oxidative Toxicity (OxT) of Oxidizer Solutions Under Exposure to Fullerenols

Bioluminescence kinetics of the enzymatic system was studied in solutions of the model organic oxidizer, 1,4-benzoquinone. Induction periods were measured in the absence and presence of fullerenols, $(T_{0.5}^{}{)}_{Ox}^{}$ and ${\left(T_{0.5}^{}\right)}_{Ox+F}^{}$, respectively. Detoxification coefficients *D_OxT_* were calculated according to Equation (3).

**Figure 5:**
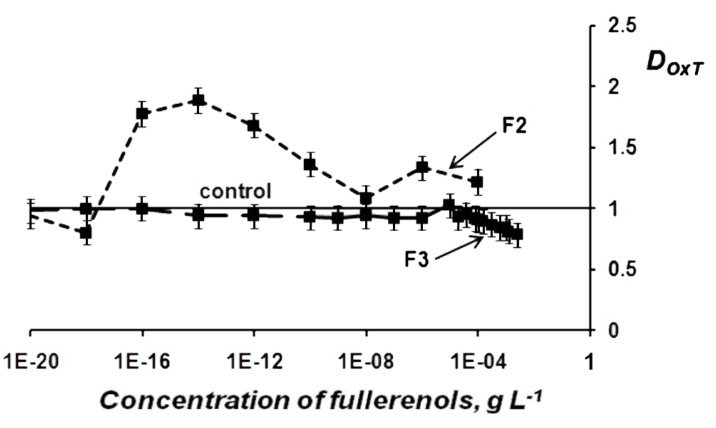
Detoxification coefficients *D_OxT_* vs. concentration of fullerenols F2 and F3 in solutions of 1,4-benzoquinone (10^−4^ M) for the enzyme-based assay.

Figure 5 demonstrates the dependence of *D_OxT_* on fullerenol concentrations. Detoxification coefficients *D_OxT_* in the solutions of organic oxidizer, 1,4-benzoquinone, reached 1.9 for F2 and were close to 1 at all F3 concentrations.

The maximal values of *D_OxT_* of fullerenols F1, F2, F3, and F4 are presented in Table 4. Similar to *D_GT_* (Table 3), the *D_OxT_* values of F1 and F2 were higher than these of F3 and F4, revealing higher antioxidant ability of fullerenols with lower oxygen numbers.

It should be noted that Figure 3, Figure 4 and Figure 5 did not demonstrate monotonic linear dependencies of the detoxification coefficients on fullerenol concentrations. The absence of such dependence is in accordance with hormesis toxicological model for low-concentration solutions of bioactive compounds [4].

### 2.2. ROS Content in Fullerenol Solutions—Luminol Chemiluminescence Assay

We supposed that ROS content was directly related with oxidative toxicity (*OxT*) of the solutions. Additionally, ROS contributed to general toxicity (*GT*) in a more complex way. Similar to excess of ROS, the lack of ROS can suppress bioluminescence of the enzymatic system since the peroxide compounds are involved in the bioluminescence reactions as intermediates [44]. Similar effects can take place in other (non-bioluminescence) redox enzymatic reactions in cells.

**Figure 6:**
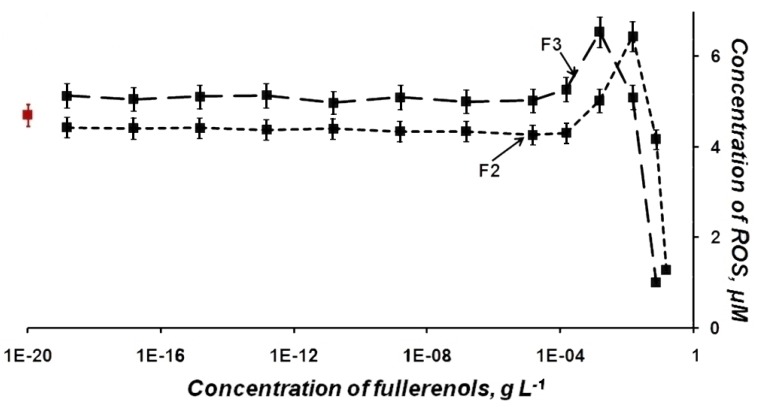
Concentration of ROS vs. concentrations of fullerenols F2 and F3 for the chemiluminescence assay. Content of ROS in distilled water is indicated by a red point in the left part of figure.

We used the luminol chemiluminescence method to compare the content of ROS in the solutions of fullerenols F1, F2, F3, and F4. Dependencies of ROS content on fullerenol concentrations were studied. Fullerenols F2 and F3 were chosen as examples for presentation in Figure 6. 

Table 5 presents the fullerenol effective concentrations, which reduced ROS content by 50%, *EC*_50_. The Table shows that F1 and F2 suppressed ROS less effectively than F3 and F4 (values of *EC*_50_ were 0.179 and 0.124 g L^−1^ as compared to 0.056 and 0.105 g L^−1^, respectively).

Inhibition and activation of bacterial bioluminescence intensity by ROS were reported previously for bacterial and enzymatic assays [59,60] where hydrogen peroxide was applied by the authors as a representative of ROS.

Correlations between characteristics of chemiluminescence and bioluminescence assays (Table 5 and Table 2, Table 3 and Table 4, respectively) were studied to confirm involvement of ROS in toxic and antioxidant effects of fullerenols F1–F4. Values of *EC*_50_, *D_GT_*, and *D_OxT_* were used to calculate correlation coefficients. The correlation coefficient between *EC*_50_ values in the chemiluminescence assay (Table 5) and the bioluminescence cellular assay (Table 2) was determined to be 0.88, while the correlation coefficient between the chemiluminescence assay (Table 5) and the bioluminescence enzymatic assay (Table 2) was 0.83. Hence, both cells and enzymes demonstrated correlations between bioluminescence intensity and ROS content. These results supported the hypothesis that ROS involvement in the bioluminescence inhibition by the fullerenols, resulted in toxic effects of these compounds in high-concentration solutions. 

The correlation coefficients between *EC*_50_ values in the chemiluminescence assay (Table 5) and detoxification coefficients *D_GT_* and *D_OxT_* in the enzyme-based assay system (Table 3 and Table 4) were 0.83 and 0.91, respectively. These results supported the suggestion of the involvement of ROS on the antioxidant effect of fullerenols in the enzyme process. This effect took place under low concentrations of fullerenols. The correlation coefficient between the *EC*_50_ values in the chemiluminescence assay (Table 5) and the values of *D_GT_* obtained using the bioluminescence cellular assay (Table 3) was 0.13. This value does not demonstrate a dependency on the ROS content, probably due to the complicated structure of the cellular assay (as compared to the enzymatic one). 

Our results showed that toxicity of fullerenols in high-concentration solutions might be related to their extra ability to neutralize oxygen radicals. Fullerenols with higher number of oxygen substituents (F3 and F4) suppressed ROS more effectively, producing more toxic effects on cellular and enzymatic systems. The antioxidant activity of fullerenols in the low-concentration solutions was probably related to their ability to regulate ROS content reversibly; fullerenols with lower number of hydroxyl substituents (F1 and F2) were characterized by higher antioxidant activity. The results might predict a higher antioxidant activity of non-substituted fullerenes, i.e., carbon nanostructures with holistic π-system apportioned evenly over the spherical macromolecule. Additional experiments under similar conditions should be performed to confirm this suggestion. Previously [61,62,63,64], the biological activity of hydrated C_60_ fullerene was studied and its activity was attributed to specific structure of hydrated shell of the fullerene. 

## 3. Materials and Methods

### 3.1. Preparations of Fullerenols

F1 and F2 were produced by fullerene hydroxylation in nitric acid followed by the hydrolysis of the polynitrofullerenes [65,66,67,68]. Preparation of F2 involved 60% of C_60_O_y_(OH)_x_ and 40% of C_70_O_y_(OH)_x_. Fullerenes were preliminary synthesized by carbon helium high-frequency arc plasma at atmospheric pressure [67,69]. The carbon soot included 12.6% of fullerene. The fullerene mixture was extracted by toluene. Then, the individual fullerene C_60_ was isolated by liquid chromatography based on turbostratic graphite with 3.42 Å interplanar distance (as a stationary phase) and a toluene/hexane mixture (as a mobile phase).

F3 was produced from a powder mixture of fullerene soot and acetylacetonate FeIII (Fe(acac)_3_). The mixture was heated up to spontaneous ignition at 180 °C. Then, the combustion process proceeded without additional heating. The product of the combustion reaction was exposed to boiling hydrochloric acid and the dissolved part of the product was removed. Retreatment with acid was provided to remove the metal salt. The solid residue of fullerenes was washed with water and used as a precursor in the synthesis of polyhydroxylated fullerenes (fullerenols). F3 was produced by precursor hydroxylation in nitric acid followed by the hydrolysis of the polynitrofullerenes [65,66,67,68].

F4 (two molecules of C_60_-fullerenol are combined by an iron atom) was produced from the powder which involved Fe-containing C_60_ fullerene soot and acetylacetonate of FeIII (Fe(acac)_3_). This mixture was heated up to spontaneous ignition (180 °C); then the temperature increased up to 250 °C in the smoldering regime. The product was treated with concentrated nitric acid (90 °C). The red cinnamonic solution was evaporated and treated by distilled water. The procedure provided the hydrolysis of poly-nitro-fullerene to poly-hydroxylated fullerene [65,66,68].

F5 (gadolinium atom inside C_82_-fullerenol) was produced by Gd@C_82_-fullerene hydroxylation in nitric acid followed by hydrolysis of the polynitrofullerenes [65,66,68,70]. Mixture of fullerenes, involving Gd@C_82_-fullerene, was preliminary synthesized by carbon helium high-frequency arc plasma at 98 kPa [67,70]. The Gd@C_82_-fullerene content in carbon soot was about 4.8%. The reaction of complexation with Lewis acids (TiCl_4_) was used for enrichment of the extract of fullerene mixture by endohedral metallofullerenes (Gd@C_82_) [71]. Then, Gd@C_82_ was extracted with carbon disulfide from carbon soot.

The fullerenol preparations were characterized with IR and photoelectron spectroscopies [72,73]. 

### 3.2. Bioluminescence Assay Systems and Experimental Data Processing

Antioxidant activity and toxicity of fullerenols were evaluated using bioluminescence assay systems, both cellular and enzymatic: (1) bacterial assay, i.e., Microbiosensor 677F, was based on the lyophilized luminous bacteria *Photobacterium phosphoreum* from the collection of the Institute of Biophysics SB RAS (CCIBSO 863), strain 667F IBSO, and (2) enzyme preparation was based on the coupled enzyme system NADH:FMN-oxidoreductase from *Vibrio fischeri* (0.15 a.u.) and luciferase from *Photobacterium leiognathi*, 0.5 mg/mL [74]. All the biological preparations were produced at the Institute of Biophysics SB RAS (Krasnoyarsk, Russia).

Chemicals used were: NADH from ICN, USA; FMN and tetradecanal from SERVA, Germany; 1,4-benzoquinone from Aldrich, USA; and sodium chloride (NaCl) from Khimreactiv, Russia.

Antioxidant activity of fullerenols was assessed in water solutions of model oxidizer, 1,4-benzoquinone.

To construct the enzymatic assay system, we used 0.1 mg ml^−1^ enzyme preparation, 4 × 10^−4^ M NADH, 5 × 10^−4^ M FMN, and 0.002% tetradecanal solutions. The enzymatic assay was performed in 0.05 M phosphate buffer, pH 6.8, at 20 °C.

The enzymatic assay system is based on two coupled enzymatic reactions:

(reaction 1) $$NADH+FMN\overset{\;NADH:FMN–oxidoreductase\;}{\to }FMN\cdot H^{-}+NAD^{-}\;\mathrm{and}$$

(reaction 2) $$FMN\cdot H^{-}+RCHO+O_{2}\overset{\;luciferase\;}{\to }FMN+RCOO^{-}+H_{2}O+hv.$$

Measurements of bioluminescence intensity were carried out with bioluminometers BLM-3606 (Nauka Special Design Bureau, Russia) and TriStar LB 941 (Berthold Technologies, Germany).

Toxic effects of fullerenols on bioluminescence of bacterial and enzymatic assay systems were characterized by relative bioluminescence intensity, $I_{F}^{rel}$:

(1) $$I_{F}^{rel}=I_{F}/I_{contr},$$

where, $I_{contr}$ and $I_{F}$ are maximal bioluminescence intensities in the absence and presence of fullerenols, respectively. 

To compare toxic effects of fullerenols, their effective concentrations that inhibited bioluminescence intensity by 50% ($I_{F}^{rel}$ = 0.5), *EC*_50_, were determined.

**Figure 7:**
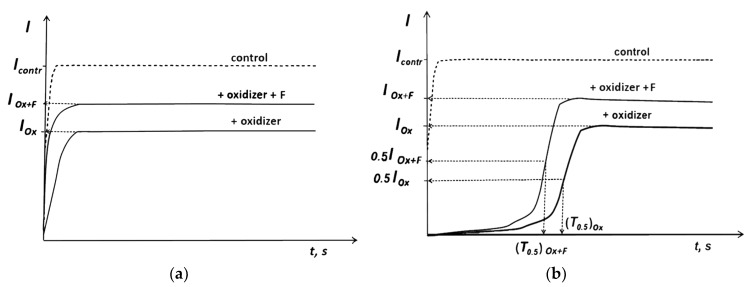
Bioluminescence kinetics in the solution of model oxidizer 1,4-benzoquinone (Ox) and fullerenol (F): (**a**) cellular assay and (**b**) enzymatic assay.

General toxicity (*GT*) of the model oxidizer solutions (1,4-benzoquinone) was evaluated with relative bioluminescence intensity, $I_{Ox}^{rel}$:

(1a) $$I_{Ox}^{rel}=I_{Ox}/I_{contr},$$

where, $I_{contr}$ and $I_{Ox}$ are maximal bioluminescence intensities in the absence and presence of the oxidizer, respectively, as shown in Figure 7. The effective concentration of model organic oxidizer (1,4-benzoquinone) that inhibited bioluminescence intensity by 50% ($I_{Ox}^{rel}$ = 0.5), *EC*_50_, were determined using bacterial and enzymatic bioluminescence assays. The *EC*_50_ values of 1,4-benzoquinone were 2.5 × 10^−7^ M and 10^−4^ M for bacterial and enzymatic assays, respectively. The values were close to those determined in previous studies [49,52].

Antioxidant activity of fullerenols was evaluated in model solutions of oxidizer (1,4-benzoquinone). *EC*_50_ of the oxidizer was used in these experiments. To exclude the peculiar toxic effects of the fullerenols, concentration ranges (CR) of the fullerenols inhibiting the bioluminescence intensity less than 10% ($I_{F}^{rel}$ > 0.9) were preliminary determined and used in the experiments. 

Both bioluminescent assays (bacterial and enzymatic) were applied to study changes in general toxicity (*GT*) under the addition of fullerenols. Detoxification coefficients *D_GT_* were determined as follows:

(2) $$D_{GT}=I_{Ox+F}^{rel}/I_{Ox}^{rel},$$

where $I_{Ox}^{rel}$, $I_{Ox+F}^{rel}$ are relative bioluminescence intensities in oxidizer solutions at *EC*_50_, in the absence and presence of fullerenols, respectively, calculated according to Equation (1a). Values of *D_GT_* were determined at different fullerenol concentrations.

To characterize oxidative toxicity (*OxT*) in the oxidizer solutions, the bioluminescence enzyme assay was used. Changes of *OxT* under fullerenol exposure were characterized with detoxification coefficients, *D_OxT_*:

(3) $$D_{OxT}={\left(T_{0.5}\right)}_{Ox}^{}/{\left(T_{0.5}\right)}_{Ox+F}^{},$$

where $(T_{0.5}^{}{)}_{Ox}^{}$ and ${\left(T_{0.5}^{}\right)}_{Ox+F}^{}$ are bioluminescence induction periods in oxidizer solutions in the absence and presence of fullerenols, respectively (Figure 7b). The *D_OxT_* values were determined and plotted vs. fullerenol concentrations.

Values of *D_GT_* > 1 or *D_OxT_* > 1 revealed a decrease in *GT* or *OxT* under the exposure to fullerenols, i.e., detoxification of solutions of oxidizers. Values of *D_GT_* ≈ 1 or *D_OxT_* ≈ 1 revealed an absence of the fullerenol effect.

The SD values for *D_GT_* or *D_OxT_* did not exceed 0.1. The data for the *D_GT_* or *D_OxT_* processing were obtained in three experiments with five samplings from all control and fullerenol solutions.

It should be noted that all experiments with ’colored’ solutions of fullerenols excluded the effect of the “optic filter” [29]; this effect did not skew the results the toxicological measurements.

### 3.3. Luminol Chemiluminescence Assay

Luminol was obtained from Sigma-Aldrich, potassium hydroxide from Khimreactiv (Russia), and 3% solution of hydrogen peroxide from Tula Pharmaceutical Factory (Russia). The 10^−4^ M aqueous alkaline luminol solution was used.

The chemiluminescence luminol reaction was initiated by a solution of K_3_[Fe(CN)_6_] and maximal value of chemiluminescence intensity was determined. All measurements were carried out in 25–40 replicates using TriStar LB 941 bioluminometer with injector system. Average and SD values did not exceed 0.05.

The dependence of chemiluminescence intensity on H_2_O_2_ concentration was initially determined and it was used as a calibration dependent in the following experiments to evaluate concentrations of peroxide compounds in the solutions of fullerenols. Peroxides were considered to be components of the ROS group. The ROS content was plotted vs. concentrations of fullerenols.

To compare the effects of fullerenols on ROS content, their effective concentrations that decreased chemiluminescence intensity by 50%, *EC*_50_, were determined. 

## 4. Conclusions

The current paper analyzed toxicity and antioxidant activity of a series of fullerenols studied under comparable conditions. In summary, all the fullerenols inhibited bacterial and enzymatic bioluminescence at high concentrations (>0.01 g L^−1^, Table 2), producing a toxic effect, while the antioxidant activity of all the fullerenols was evident at low and ultralow concentrations (<0.001 g L^−1^, Table 2). We found that the toxic and antioxidant characteristics of the fullerenols depended, additionally, on the number of oxygen substituents. Quantitative characteristics of the fullerenols (effective concentrations, concentration ranges, and detoxification coefficients) were determined and compared. 

Lower toxicity and higher antioxidant activity were demonstrated for the fullerenols with fewer substituents: C_60_O_y_(OH)_x_ and C_60,70_O_y_(OH)_x_, where x+y = 24–28. The differences were attributed to fullerenol’ ability to disturb ROS balance in aqueous solutions. Further investigations, including theoretical studies, should be carried out to understand the physical and chemical basis of these differences. The investigations should be aimed at such structural fullerenol peculiarities as an interrelation between the number of oxygen-containing groups and hydrophobic π-conjugated surface fragments, with the latter responsible for the reversible electron acceptance and, hence, nonspecific catalytic activity in chemical and biochemical processes. 

As an outlook, a recommendation can be made for the selection and synthesis of fullerene’ water-soluble derivatives: a high number of oxygen substituents (up to 40 and more) provided higher toxicity and lower antioxidant activity. 

Hence, the study demonstrated a suitability and high potential for the bioluminescence-based biosensing procedure for the complex study of the carbon nanoparticles, which are promising pharmaceutical agents.

## Figures and Tables

**Table 1:** Fullerenol’ chemical formulas and short abbreviations.

Chemical Formula	x+y	Abbreviation
C_60_O_y_(OH)_x_	24–28	F1
C_60,70_O_y_(OH)_x_	24–28	F2
C_60,70_O_y_(OH)_x_	40–42	F3
Fe_0,5_C_60_O_y_(OH)_x_	40–42	F4
Gd@C_82_O_y_(OH)_x_	40–42	F5

**Table 2:** Values of effective concentrations EC_50_ of fullerenols F1–F5 and concentration range (CR) of F1–F5 for decreasing bioluminescent intensity less than 10% for both enzymatic and cellular assays.

Fullerenols	Cellular Assay		Enzymatic Assay	
	Fullerenol Concentration (g L^−1^)			
	EC_50_	CR	EC_50_	CR
F1	0.031	<0.010	0.092	<0.010
F2	0.008	<0.002	0.021	<0.010
F3	0.003	<0.001	0.007	<0.003
F4	0.021	<0.012	0.007	<0.001
F5	-	-	0.018	<0.005

**Table 3:** Maximal values of *D_GT_* of fullerenols (F1–F4) in model solutions of organic oxidizer (1,4-benzoquinone) for enzymatic and cellular assays.

Fullerenols	Maximal Value of *D_GT_*	
	Enzymatic Assay	Cellular Assay
F1	2.2	1.4
F2	1.5	1.8
F3	1.3	1.3
F4	1.1	1.3

**Table 4:** Maximal values of *D_OxT_* of fullerenols (F1–F4) in model solutions of organic oxidizer (1,4-benzoquinone) for the enzymatic assay.

Fullerenols	Maximal Value of *D_OxT_*
F1	2.0
F2	1.9
F3	1.0
F4	1.3

**Table 5:** Values of *EC*_50_ of fullerenols in chemiluminescence assay.

Fullerenols	*EC*_50_, g L^−1^
F1	0.179
F2	0.124
F3	0.056
F4	0.105

## Abbreviations

CR	concentration range
F	fullerenol
F1	fullerenol C_60_O_y_(OH)_x_, where x+y = 24–28
F2	fullerenol C_60,70_O_y_(OH)_x_, where x+y = 24–28
F3	fullerenol C_60__,70_O_y_(OH)_x_, where x+y = 40–42
F4	fullerenol Fe_0,5_C_60_O_y_(OH)_x_, where x+y = 40–42
F5	fullerenol Gd@C_82_O_y_(OH)_x_, where x+y = 40–42
FMN	flavin mononucleotide
GT	general toxicity
NADH	nicotinamide adenine dinucleotide disodium salt-reduced
OxT	oxidative toxicity
ROS	reactive oxygen species
